# Supplementary material for: traceax: a JAX-based framework for stochastic trace estimation
Source: bioRxiv. 2025 Jul 20:2025.07.14.662216. Preprint. [Version 2] doi: 10.1101/2025.07.14.662216 (PMC12338724; doi:10.1101/2025.07.14.662216)
Supplement: Supplement 2 [file NIHPP2025.07.14.662216v2-supplement-2.pdf]

bioRxiv preprint doi: https://doi.org/10.1101/2025.07.14.662216; this version posted July 20, 2025. The copyright holder for this preprint (which was not certified by peer review) is the author/funder, who has granted bioRxiv a license to display the preprint in perpetuity. It is made available under aCC-BY 4.0 International license.

traceax: a JAX-based framework for stochastic trace estimation

Abdullah Al Nahid[1], Linda Serafin[3], Nicholas Mancuso[2,3,4]

1. Alfred E. Mann School of Pharmacy and Pharmaceutical Sciences, University of Southern California, Los Angeles, CA, USA
2. Center for Genetic Epidemiology, Keck School of Medicine, University of Southern California, Los Angeles, CA, USA
3. Department of Population and Public Health Sciences, Keck School of Medicine, University of Southern California, Los Angeles, CA, USA
4. Department of Quantitative and Computational Biology, University of Southern California, Los Angeles, CA

bioRxiv preprint doi: https://doi.org/10.1101/2025.07.14.662216; this version posted July 20, 2025. The copyright holder for this preprint (which was not certified by peer review) is the author/funder, who has granted bioRxiv a license to display the preprint in perpetuity. It is made available under aCC-BY 4.0 International license.

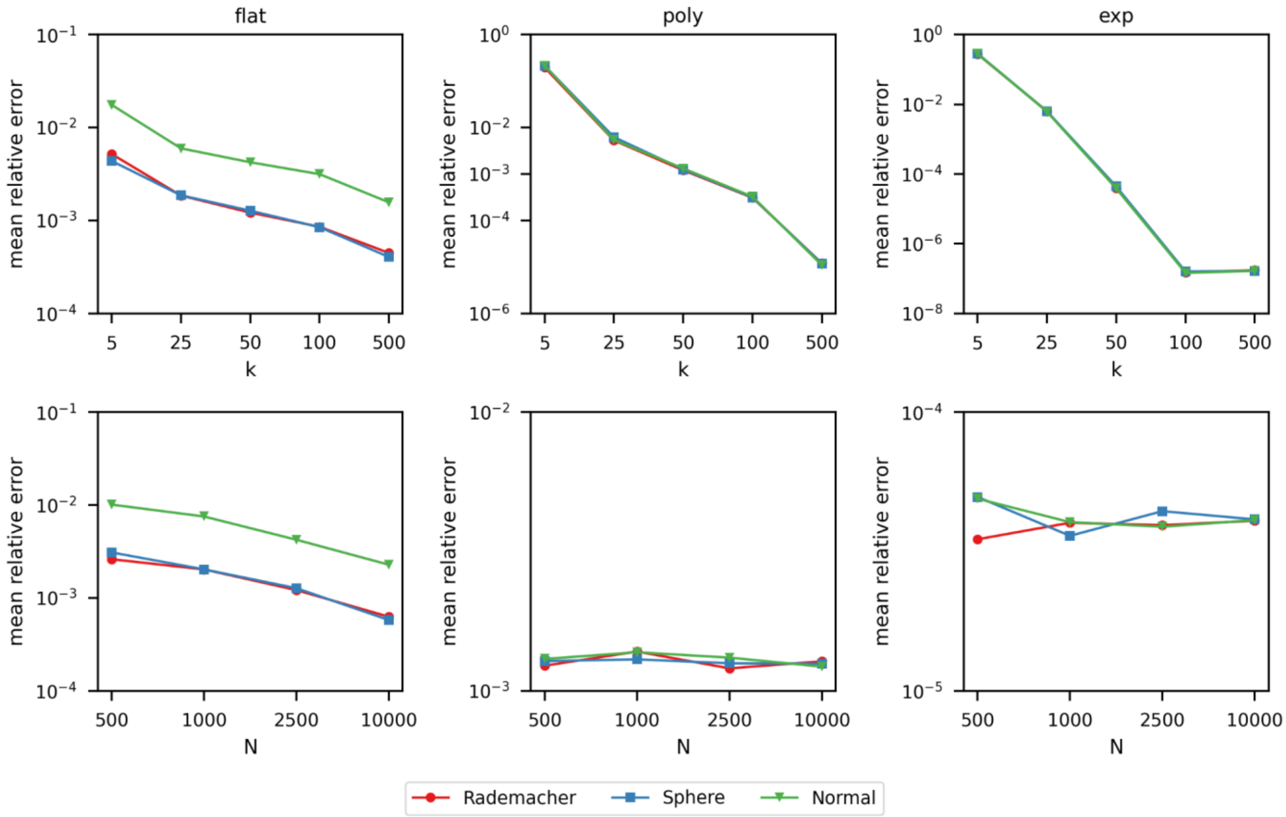

Figure S1. Mean relative error of XTrace with different probe distributions. *Top row:* mean relative error plotted against probe size k, with fixed matrix size N=2500. *Bottom row:* mean relative error plotted against N with fixed k=50. Columns correspond to different eigenvalue spectra: flat (left), polynomial decay (middle), and exponential decay (right). The three probe distributions compared are rademacher, sphere, and normal. Each setting was run 100 times.

bioRxiv preprint doi: https://doi.org/10.1101/2025.07.14.662216; this version posted July 20, 2025. The copyright holder for this preprint (which was not certified by peer review) is the author/funder, who has granted bioRxiv a license to display the preprint in perpetuity. It is made available under aCC-BY 4.0 International license.

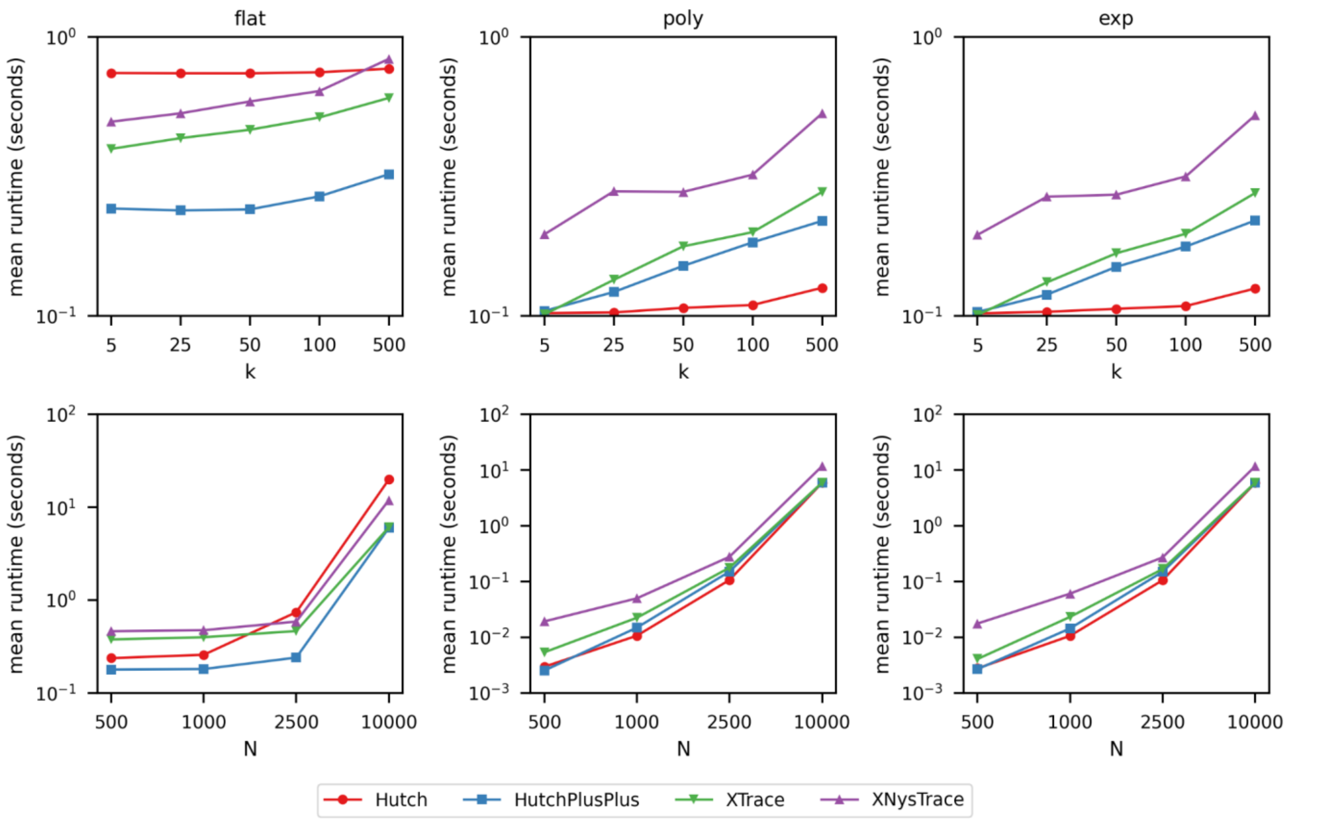

Figure S2. Runtime performance of different estimators. *Top row:* mean runtime plotted against probe size k, with fixed matrix size N=2500. *Bottom row:* mean runtime plotted against N with fixed k=50. Columns correspond to different eigenvalue spectra: flat (left), polynomial decay (middle), and exponential decay (right). The four methods compared are Hutch, Hutch++, XTrace, and XNysTrace. Each scenario was run 100 times.

bioRxiv preprint doi: https://doi.org/10.1101/2025.07.14.662216; this version posted July 20, 2025. The copyright holder for this preprint (which was not certified by peer review) is the author/funder, who has granted bioRxiv a license to display the preprint in perpetuity. It is made available under aCC-BY 4.0 International license.

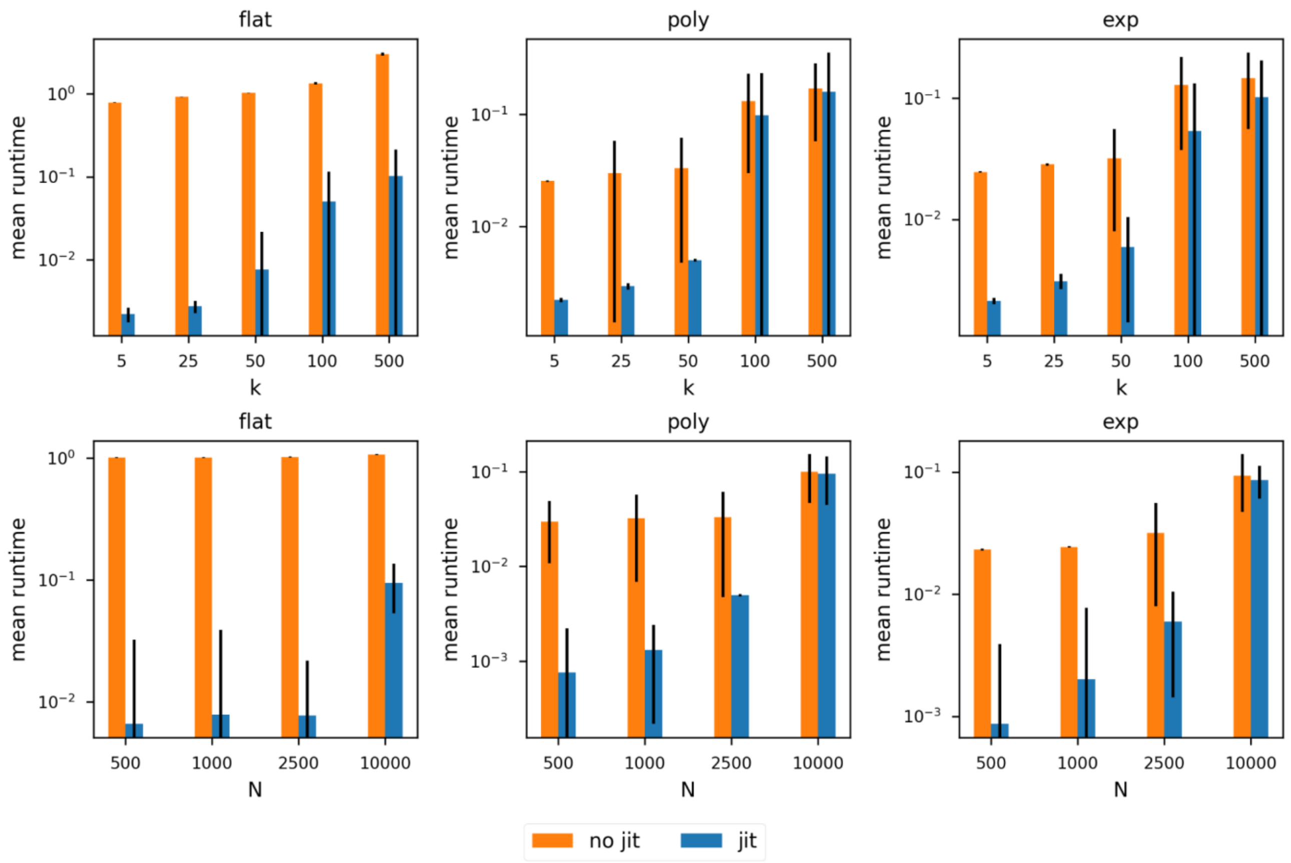

Figure S3. Mean runtime comparison of JIT and no-JIT version of XTrace estimation. *Top row:* mean runtime plotted against probe size k, with fixed matrix size N=2500. *Bottom row:* mean runtime plotted against N with fixed k=50. Columns correspond to different eigenvalue spectra: flat (left), polynomial decay (middle), and exponential decay (right). Orange bars indicate no JIT compilation, whereas blue bars indicate JIT compiled trace estimation. Each setting was run 100 times.

bioRxiv preprint doi: https://doi.org/10.1101/2025.07.14.662216; this version posted July 20, 2025. The copyright holder for this preprint (which was not certified by peer review) is the author/funder, who has granted bioRxiv a license to display the preprint in perpetuity. It is made available under aCC-BY 4.0 International license.

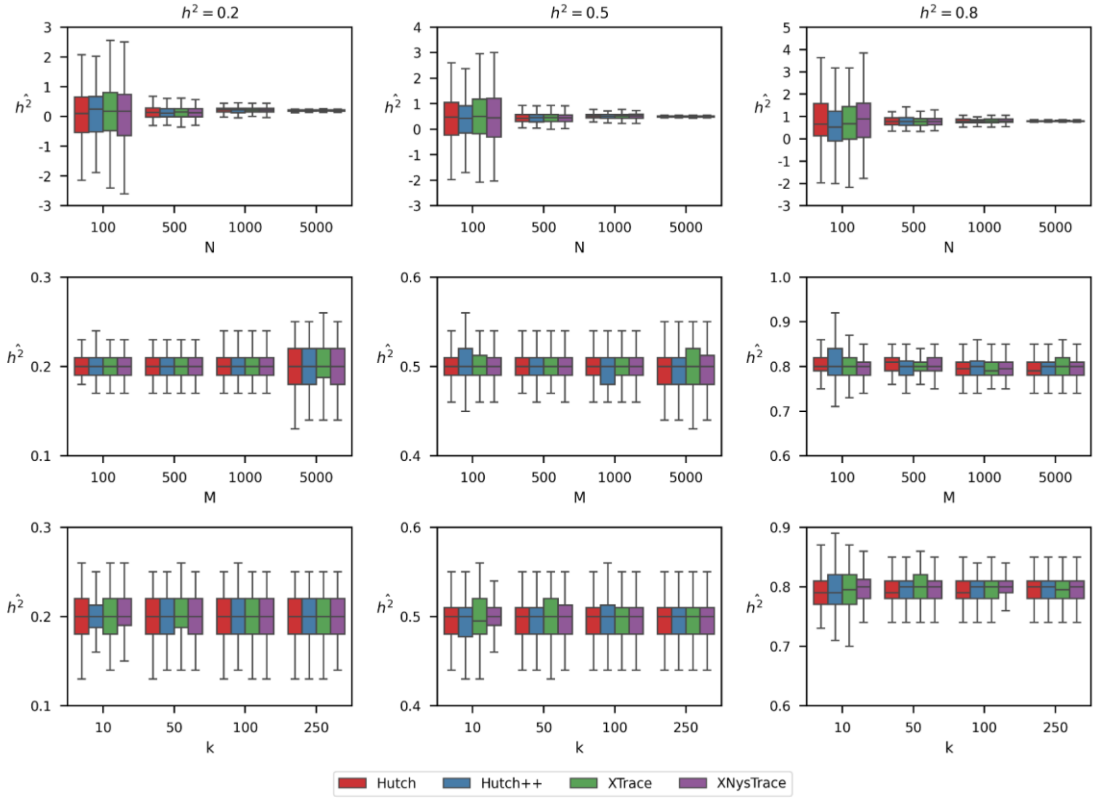

Figure S4. Benchmarking estimators for recovering h^2 with the RHE-reg heritability estimator using traceax. *Top row:* h^2 estimates plotted against sample size N, with fixed number of SNPs M=5000 and probe size k=50. *Middle row:* h^2 estimates plotted against M, with fixed N=5000 and k=50. *Bottom row:* h^2 estimates plotted against k, with N=5000 and M=5000. Columns correspond to different true heritability values: h^2=0.2 (left), h^2=0.5 (middle), and h^2=0.8 (right). The four methods compared are Hutch, Hutch++, XTrace, and XNysTrace. Each configuration was run 100 times with default parameters unless otherwise stated.

bioRxiv preprint doi: https://doi.org/10.1101/2025.07.14.662216; this version posted July 20, 2025. The copyright holder for this preprint (which was not certified by peer review) is the author/funder, who has granted bioRxiv a license to display the preprint in perpetuity. It is made available under aCC-BY 4.0 International license.

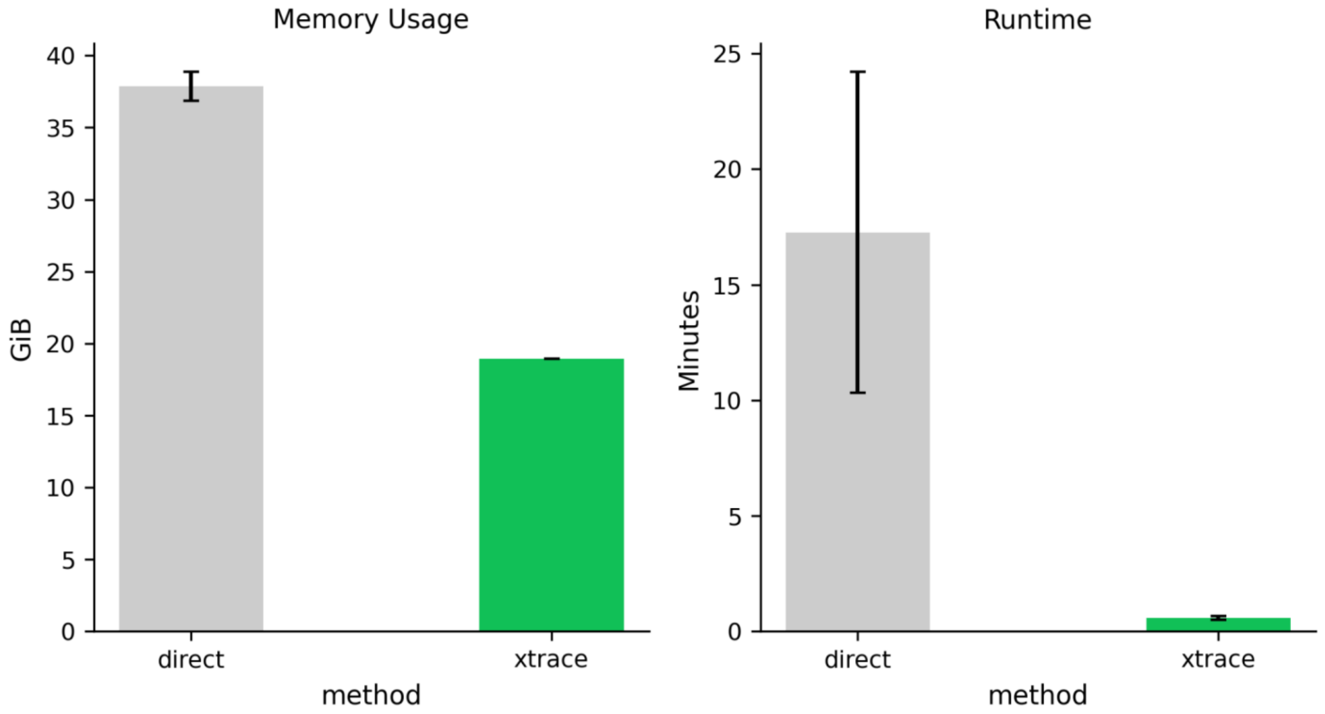

Figure S5. Memory usage and runtime comparison of RHE-reg SNP heritability estimation using direct vs XTrace trace computation. Across 10 independent runs, memory usage and runtime were evaluated for the RHE-reg SNP heritability estimator, comparing direct trace computation with the XTrace estimator implemented in *traceax*. Each run used N=50,000 individuals, M=50,000 SNPs, k=100 random probe vectors, and true heritability $\boldsymbol{h^2}$=0.5. Both methods produced accurate $\boldsymbol{h^2}$ estimates.
